# Serially measured urinary albumin excretion is strongly associated with incident heart failure with preserved ejection fraction

**DOI:** 10.1002/ejhf.3739

**Published:** 2025-07-07

**Authors:** Thomas F Kok, Bart J. van Essen, Yiqian Yang, Ron T. Gansevoort, Stephan J.L. Bakker, Kevin Damman, Eric Boersma, Isabella Kardys, Adriaan A. Voors, Rudolf A. de Boer, Navin Suthahar

**Affiliations:** ^1^ Department of Cardiology Erasmus MC, Cardiovascular Institute, Thorax Center Rotterdam The Netherlands; ^2^ Department of Cardiology University Medical Center Groningen Groningen The Netherlands; ^3^ Division of Nephrology, Department of Internal Medicine University Medical Center Groningen, University of Groningen Groningen The Netherlands

## Introduction

Increased urinary albumin excretion (UAE) is not only a marker of kidney disease but also a strong risk factor for heart failure (HF).^1^ From a pathophysiologic standpoint, elevated UAE reflects systemic endothelial dysfunction (i.e. increased endothelial permeability)—a key mechanism implicated in HF pathogenesis.

Elevated UAE is frequently observed in HF patients, and it is nearly twice as prevalent in those with HF and preserved ejection fraction (HFpEF) compared to those with HF and reduced ejection fraction (HFrEF) (approximately 40% vs. 24%).^1^ Nevertheless, in prospective studies, UAE measured at a single time point is strongly associated with incident HF—with no substantial differences between HF subtypes.^2^

A recent study examining longitudinal UAE trajectories found that persistently high UAE levels were significantly associated with increased HF risk^3^; however, it did not distinguish between HF subtypes. To our knowledge, no prior studies have specifically examined the relationship between serial UAE measurements and incident HFpEF and HFrEF, separately. Distinguishing between HF subtypes is important because temporal increases in UAE likely reflect progressive endothelial dysfunction—a process considered central to HFpEF pathophysiology.

In this context, we aimed to investigate the relationship between serial UAE and incident HF subtypes. We hypothesized that serially measured UAE would be more strongly associated with the risk of developing HFpEF than HFrEF.

## Methods

The PREVEND study is a Dutch general population cohort consisting of 8592 participants.^4^ The PREVEND study was approved by the local medical ethics committee of the University of Medical Center Groningen (approval number: MEC96/01/022) and conformed to the principles drafted in the Helsinki Declaration. For the current study, we excluded participants with prevalent HF (*n* = 23), unavailable left ventricular ejection fraction (*n* = 11), unavailable UAE data (*n* = 2), or missing adjustment variables (*n* = 218) resulting in a final study population of 8338 participants, of which 4169 (50.0%) were women.

Urinary albumin excretion was measured at baseline and at four additional time points after a median of 4.2, 6.4, 9.3, and 11.7 years, respectively. HF was assessed by an endpoint adjudication committee, who checked patient files from the two main hospitals in the region of Groningen, The Netherlands. Further details are described elsewhere.^4^ Follow‐up lasted from baseline visit (1997–1998) until participants died, developed HF, moved away, or reached the censoring date (1 January 2022).

We estimated temporal trends in UAE using linear mixed‐effects models. The time difference (in years) between time of UAE measurement and time of outcome or censoring was used as the timescale and modelled using cubic splines for fixed effects, and using both random intercepts and random slopes for random effects. Trajectories were generated for participants without incident HF, with incident HFpEF, and with incident HFrEF.

Joint models for longitudinal and time‐to‐event data were used to investigate associations of serial UAE measurements with incident HFrEF and HFpEF. Joint models combine a linear mixed‐effects model, which describes the trajectory of a predictor, with a time‐to‐event relative risk model to relate the estimated temporal pattern of a predictor with the hazard of the outcome of interest. The joint models were estimated using a Bayesian approach, using Markov chain Monte Carlo algorithms, to calculate hazard ratios (HRs) and 95% confidence intervals (CIs). To account for the skewed data distribution, UAE values were log‐2 transformed and the HRs represent the effect per doubling of UAE levels at any point during follow‐up, between two participants. Effect size differences between HFrEF and HFpEF were assessed using a Z‐score derived from HRs and their standard deviations. Statistical analyses were performed using R version 4.2.2.

## Results

**Figure 1:**
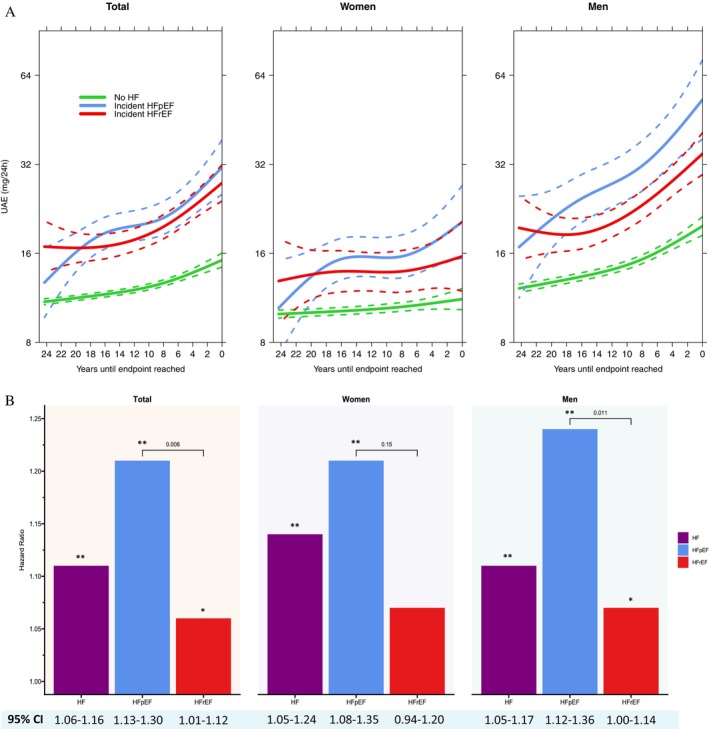
(*A*) Temporal trends in urinary albumin excretion (UAE) according to incident heart failure (HF) with preserved (HFpEF) and reduced ejection fraction (HFrEF). Temporal trends in UAE were estimated using a linear mixed‐effects model, incorporating cubic splines for fixed effects. UAE was measured in 8324 participants (99.8%) at baseline, 6618 (79.4%) at visit 2, 5663 (67.9%) at visit 3, 4833 (58.0%) at visit 4, and 4369 (52.4%) at visit 5. The *x*‐axis represents the number of years between each UAE measurement and the time of HFpEF diagnosis, HFrEF diagnosis, or censoring. Time 0 (rightmost on the *x*‐axis) marks the time of occurrence of HFpEF, HFrEF, or censoring. All UAE measurements occurred at varying moments prior to Time 0; increasing values to the left indicate earlier UAE measurement times. Despite unbalanced follow‐up and variation in UAE availability across visits, the model estimates average temporal UAE trends by incorporating data from all available time points across all participants. The predicted trajectories suggest that elevated UAE precedes both incident HFpEF and HFrEF by approximately 20 years. (*B*) Associations of serial UAE measurements with incident HFpEF and HFrEF. Joint models were used to associate serially measured UAE with outcome. Hazard ratios reflect the relative risk of a doubling in UAE levels, at any time point during follow‐up, between two participants. Models were adjusted for age, relative fat mass, systolic blood pressure, blood glucose, cholesterol, smoking status, antihypertensive medication use, history of myocardial infarction, cerebrovascular event, and atrial fibrillation. For analyses in the total population, models were additionally adjusted for sex. Bars describe effect sizes for HF, HFpEF and HFrEF. CI, confidence interval. *p ≤ 0.05; **p ≤ 0.001.

During 156 239 person‐years of follow‐up (median 23.4 years; P25–P75: 13.1–23.8 years), 533 HFrEF and 263 HFpEF events occurred. Temporal trends in UAE, stratified by incident HFrEF, incident HFpEF, or absence of HF, are displayed in *Figure* *1A*. In joint models, doubling of UAE was strongly associated with an increased risk of incident HF (HR 1.11; 95% CI 1.06–1.16), with a stronger effect size for HFpEF (HR 1.21; 95% CI 1.13–1.30) than HFrEF (HR 1.06; 95% CI 1.01–1.12) (*p*_difference_ = 0.008) (*Figure* *1B*). In sex‐specific analyses, numerical trends were broadly similar although a significant difference between HF subtypes (HFpEF vs. HFrEF) was only found in men.

## Discussion

In the current study we hypothesized that serially measured UAE in the general population would be more strongly associated with incident HFpEF than incident HFrEF. We found that over a follow‐up of 25 years, UAE trajectories were higher among individuals developing HFpEF than HFrEF, and serially measured UAE showed a stronger association with the risk of developing HFpEF than HFrEF.

These findings are relevant because the prevalence of HFpEF continues to rise rapidly, while current treatment options remain limited, primarily to sodium–glucose cotransporter 2 (SGLT2) inhibitors. Early identification of individuals at risk for developing HFpEF is crucial, as this could enable earlier implementation of lifestyle‐based therapeutic interventions or earlier initiation of SGLT2 inhibitors—potentially reducing the overall burden of HFpEF in the community. Based on our data, serially measured UAE appears to be a promising tool for early identification of HFpEF, although prospective validation studies are warranted.

Study strengths include: (i) this is the first study to examine associations of serial UAE with HFpEF and HFrEF; (ii) in the PREVEND study, UAE was measured using 24‐h urine collection, which is the gold standard to assess albuminuria; and (iii) the follow‐up duration was 25 years and all HF cases were adjudicated. Study limitations include: (i) as the PREVEND population is predominantly Caucasian, our findings should be validated in other ethnicities/races; and (ii) we acknowledge that, although our study included serial UAE measurements, causality cannot be inferred from observational data. Nonetheless, our findings complement initiatives like the THOMAS (Towards HOMe‐based Albuminuria Screening) trial^5^ and highlight the need for further research to evaluate the role of serial UAE monitoring in HFpEF assessment.

## Conclusion

Serially measured UAE is more strongly associated with the development of HFpEF than HFrEF.

### Funding

Drs. Suthahar and de Boer are supported by the Netherlands Heart Foundation (Hartstichting) through grants 2020B005 (CVON DOUBLE DOSE) and 01‐003‐2022‐0358 (CARMA), by the Netherlands Organization for Scientific Research (NWO), co‐funded by ERA4Health through the CARDINNOV 2023 caLL, as part of the EnerLIGHT project (Grant Agreement No. 101095426 of the EU Horizon Europe Research and Innovation Programme) and by the European Research Council (ERC CoG 818715; SECRETE‐HF). The PREVEND study was financially supported by grant E.013 from the Dutch Kidney Foundation (Nierstichting).

**Conflict of interest**: K.D. reports speaker and consultancy fees to his employer by Abbott, Novartis, AstraZeneca, Boehringer Ingelheim, FIRE1, Novartis, Echosense and Reprieve. I.K. received travel support from Olink and SomaLogic. The employer of A.A.V. received consultancy fees and/or research support from Adrenomed, Anacardio, AstraZeneca, Bayer AG, BMS, Boehringer Ingelheim, Corteria, Eli Lilly, Merck, Moderna, Novartis, Novo Nordisk, Roche diagnostics, SalubrisBio. R.A.d.B. has had speaker engagements, received fees, and/or served on advisory boards for Abbott, AstraZeneca, Bristol Myers Squibb, Novo Nordisk, Roche, and Zoll; and received travel support from Abbott and Novo Nordisk, all outside the present work. The institution where R.A.d.B. is employed has received research grants and/or fees from Alnylam, AstraZeneca, Abbott, Bristol‐Myers Squibb, Novo Nordisk and Roche. All other authors have nothing to disclose.
